# Methodological and ethical issues in research using social media: a metamethod of Human Papillomavirus vaccine studies

**DOI:** 10.1186/1471-2288-14-127

**Published:** 2014-12-02

**Authors:** Diana L Gustafson, Claire F Woodworth

**Affiliations:** Faculty of Medicine, Memorial University, St. John’s, Canada

**Keywords:** Metamethod, Social media, Data collection, HPV vaccination, Ethics, Methodology

## Abstract

**Background:**

Online content is a primary source of healthcare information for internet-using adults and a rich resource for health researchers. This paper explores the methodological and ethical issues of engaging in health research using social media.

**Methods:**

A metamethod was performed on systematically selected studies that used social media as a data source for exploring public awareness and beliefs about Human Papillomaviruses (HPV) and HPV vaccination. Seven electronic databases were searched using a variety of search terms identified for each of three concepts: social media, HPV vaccine, and research method. Abstracts were assessed for eligibility of inclusion; six studies met the eligibility criteria and were subjected to content analysis. A 10-item coding scheme was developed to assess the clarity, congruence and transparency of research design, epistemological and methodological underpinnings and ethical considerations.

**Results:**

The designs of the six selected studies were sound, although most studies could have been more transparent about how they built in rigor to ensure the trustworthiness and credibility of findings. Statistical analysis that intended to measure trends and patterns did so without the benefit of randomized sampling and other design elements for ensuring generalizability or reproducibility of findings beyond the specified virtual community. Most researchers did not sufficiently engage virtual users in the research process or consider the risk of privacy incursion. Most studies did not seek ethical approval from an institutional research board or permission from host websites or web service providers.

**Conclusions:**

The metamethod exposed missed opportunities for using the dialogical character of social media as well as a lack of attention to the unique ethical issues inherent in operating in a virtual community where social boundaries and issues of public and private are ambiguous. This suggests the need for more self-conscious and ethical research practices when using social media as a data source. Given the relative newness of virtual communities, researchers and ethics review boards must work together to develop expertise in evaluating the design of studies undertaken with virtual communities. We recommend that the principles of concern for welfare, respect for person, and justice to be applied in research using social media.

## Background

Social media, such as Twitter, Facebook and YouTube, are changing how people build relationships, share information, and make everyday decisions, including decisions about health and wellbeing [1–3]. According to *The Social Life of Health Information*, *2011 Report* by Pew Internet, 80% of internet-using adults have consulted a website as a primary source of information on healthcare topics [4]. An online Angus Reid survey commissioned by Rogers Inc. indicated that 89% of 1,010 randomly selected Canadian adults use the internet to research health issues and symptoms [5]. Likewise, the Pew Research Internet Project found that 59% of American adults looked online for health information in 2013 [6]. The percentage and frequency of use may be higher among adolescents and young adults who use social media as a primary platform for networking, communicating and information seeking [7]. Maczewski reported that blogs and other forms of social media play an increasingly significant role in youths’ development by helping them maintain social relationships, deal with continuous cultural change, and grow an emerging sense of self [7]. Thus, understanding how youth and young adults use social media to support health decision-making should be of interest to public health researchers, physicians, and other health care providers.

What fits under the umbrella of social media is getting broader rather than better defined because of the range of platforms and modes of interactivity that have rapidly evolved since the first social networking site, GeoCities, was established in 1994. For the purpose of our study we define *social media* as an electronic medium that allows interactive comment, as in the case of platforms such as Twitter and YouTube, or collaboration and information sharing, as with Pinterest and Wikipedia.^a^

Some researchers are using social media as a means of networking with others, recruiting study participants, collecting data (e.g., mining blogs or engaging with focus groups), and disseminating new knowledge. What is less clear is how health researchers design and conduct studies using social media and engage ethically with a virtual community.

This paper presents the findings of a metamethod of selected studies that used social media as a data source for exploring public awareness and beliefs about Human Papillomaviruses (HPV) and HPV vaccination. The results offer insight into how health researchers are designing and conducting such studies, the quality of data generated, the trustworthiness and credibility of results, and if and how they identified and addressed ethical issues when engaging with a virtual community. We anticipate that our findings will provoke discussion about methodological and ethical considerations when using social media as a data source for studying public perceptions about HPV vaccination and other health-related issues, especially when engaging with youth and other potentially vulnerable populations.

### What is a metamethod?

A metamethod is second-order research on the methods used to generate findings and is one component in a tri-partite meta-analysis framework [8]. Specifically, a metamethod sequentially examines the methods, methodological assumptions, and procedural rules that guide the research design and the approach to data collection and analysis across a number of similar, systematically selected primary studies [8, 9]. Evaluation of the deep structures (i.e., the epistemological assumptions, procedural strengths and limitations, and rigor in design elements) is invaluable in establishing the appropriateness and suitability of a methodological approach to a particular phenomenon [10]. Because the deep structures reflect the researchers’ epistemic stance and role in the production of new knowledge, conducting a metamethod contributes to methodological reflexivity and self-conscious research practice, ultimately shaping disciplinary trends and current understandings about specific social phenomena [11].

### Why social media and HPV vaccine studies?

There appears to be a good fit between those who use social media as a primary channel for accessing and sharing health information (not to the exclusion of other sources), and health researchers who want to track user groups and compare information concerning individual attitudes and behavior about health issues. The timing of the HPV vaccine and the rise of social media are congruent. Researchers have thus used social media as a data source to explore the public’s opinions on HPV vaccination as youth, young adults, and adults went online to express concerns and seek information about a vaccine for the first time. In view of the rapid growth of social media since 2005 and the licensing of Gardasil in 2006 it seemed prudent to carefully examine the epistemological, methodological and ethical considerations for researchers conducting HPV studies in a burgeoning environment.

Specifically, we were interested in researchers’ use of social media for exploring public perceptions of HPV vaccination so that we could design a sound research project that was likely to produce credible and trustworthy results for our local context. This paper details our reflections on the methodological assumptions and ethical issues that emerged when we conducted a systematic review of selected HPV vaccination studies. These reflections were integrated into a study DLG initiated in 2014, and may contribute more broadly to self-conscious and ethical health research practice using social media.

## Methods

### Objectives

1. To analyze primary research studies that used social media to explore knowledge about HPV infections and vaccination.
2. To clarify, synthesize, and reflect on the possibilities and ethical considerations of using social media as a data collection tool in health research.

### Selection and appraisal of primary research studies

For this metamethod, we, like others before us [11], assumed an interpretive qualitative research approach in the social constructivist paradigm. In practice, this meant that we sought to understand how health researchers were collecting data in virtual communities and analyzing and constructing knowledge about public perceptions of HPV vaccinations.

After consulting with a library scientist, we identified and searched seven subject-specific electronic databases most suitable for our purposes: Business Source Complete, Cumulative Index of Nursing and Allied Health Literature (CINAHL), Educational Resources Information Center (ERIC), PsychInfo, PubMed, Sociological Abstracts, and Women’s Studies International. A variety of search terms were identified for each of three key concepts: social media, HPV vaccine, and research method (see Table 1). All primary research studies published in English language peer reviewed journals between January 2005 (when social media became prominent) and June 2012 (when data collection commenced) were eligible for inclusion if the study used social media as a tool for collecting data about HPV vaccination.

**Table 1:** **Search terms**

Key concepts	Search terms
Social media AND	Social medi* or blog* or discussion board* or message* board* or Facebook or internet or Myspace or Twitter or Web 2.0 or web log* or Wiki* or YouTube
HPV vaccine AND	Cervirax or Gardasil or Human Papilloma Virus or HPV immuniz* or Human Papilloma Virus or HPV immunis* or Human Papilloma Virus or HPV inoculation or Human Papilloma Virus or HPV vaccin*
Research method	Data source* or research method* or data collection tool*

The *symbol refers to truncation of a search term.

The first step in our metamethod was an initial review of the abstracts of all retrieved studies to determine if they were relevant. Both authors did this review independently before discussing potentially eligible studies. Disagreements were resolved through dialogue and a list of select articles was created. Reference lists of the selected articles were hand searched by title for other potentially eligible studies and the list was finalized.

Next, we codified the procedural steps for assessing the methodological orientation and features of selected studies. Drawing on the foundational guidelines developed by Paterson and colleagues [8] and Minnaar’s [12] use of a metamethod in e-learning, we developed a 10-item coding scheme (see Table 2). Each item had a set of questions that, taken together, assessed the clarity, congruence and transparency of the research design and its epistemological and methodological underpinnings. After independently testing the coding scheme using a de-selected study, we added and clarified questions to improve the comprehensibility of the scheme and to ensure we had a common understanding of each item and related questions.

**Table 2:** **Coding scheme**

Article author/title	Guiding questions
Country	
Research question	1. Is the research question/problem clearly stated?
	2. Is the research question meaningful, significant, and worthy of investigation?
Study aim/purpose	3. Is the study aim/purpose clearly stated?
	4. Does the literature review provide sufficient context and background to justify the study?
	5. Are the concepts central to the research question clearly defined?
Design type	6. Is the methodological approach clearly stated?
	7. What methodology guides the design?
	8. Are the methodological assumptions underpinning the study clearly stated?
	9. Is there a fit between the methodological approach and the research question?
Data source	10. What is the data source?
	11. Is the data source appropriate for the research question?
Method	12. Is the data collection method clearly described?
	13. Is the data collection method appropriate for the research question?
	14. Is the sample size clearly stated?
	15. Is recruitment clearly described?
	16. Is the data collection method consistent with the methodological approach?
Data analysis	17. Is the approach to data analysis clearly stated?
	18. Is the method of data analysis consistent with the methodological approach?
Rigor	19. Evidence of credibility/internal validity: e.g., member checking; triangulation; prolonged engagement
	20. Evidence of transferability/external validity: e.g., robust description of setting, participants, content and findings
	21. Evidence of dependability/reliability: audit trail describing researcher(s) data, methods, analysis and decision-making
	22. Evidence of confirmability/objectivity: clearly stated epistemic stance including researcher assumptions, reflectivity and audit trail
Findings	23. Are the findings clearly stated?
	24. Is the interpretation of the findings well supported by the literature?
	25. Are the findings/conclusions clearly linked to the data?
Ethical issues	26. Does the study follow principles of ethical research?
	27. Did the study receive ethics approval?
Overall	28. Is the research design sound?

We used the coding scheme to independently analyze each selected study. We discussed our progress with coding on three occasions via Skype and met a fourth time in person to share our notes, address disagreements, and come to a consensus on our findings. The coding scheme (and our process of developing it) served several purposes that are key to maintaining the integrity of a metamethod. These are: (a) improved efficiency in coding a moderate amount of data; (b) better consistency in coding between coders; (c) enhanced soundness of data interpretation between coders of differing orientations and levels of experience (DLG was a social scientist with 15+ years’ experience and CFW was an undergraduate student with 1–2 years research experience in the biomedical sciences); (d) increased comprehensibility in interpretation; and (e) supported intersubjectivity and divergent meaning-making [13]. Finally, the coding scheme provided a concrete analytic tool that may be useful to other researchers who replicate this study or conduct their own metamethod in the future.

## Results

### Description of selected studies^b^

Of the 91 studies that were retrieved, 8 were duplicates and 77 were deemed not relevant, most often because a study did not use social media as a tool for collecting data about HPV vaccination.^c^ Hand searching reference lists of selected articles produced some duplicates but no additional studies. Six articles were subjected to content analysis using the coding scheme.

Studies were conducted by research teams from Canada (3) [14–16], Korea (1) [17], and United States (2) [18, 19] where HPV vaccination programs had been implemented. Three articles [16, 18, 19] presented findings of content analyses of videos and viewer-posted responses or comments posted on YouTube while a fourth article [15] presented findings of a content analysis of blogs posted on Myspace. A fifth article [17] was a longitudinal study of public and private questions posted on a physician-researcher created HPV information website. A sixth study [14] was a virtual ethnography of discussion threads in response to questions about HPV vaccination posted on a publicly accessible message board (see Table 3).

**Table 3:** **Summary of article features** ^**e**^

Article and country	Design type	Data source	Ethical considerations
Ache & Wallace [18] United States	Descriptive statistical analysis	YouTube videos Viewer posted comments	None raised
Battles [14] Canada	Virtual ethnography (cyberethnography)	Message board	Several issues raised
Briones et al. [19] United States	Descriptive statistical analysis	YouTube videos Viewer posted like/dislike	None raised
Keelan et al. [15] Canada	Content analysis and Descriptive statistical analysis	Myspace blogs Blogger characteristics	None raised
Keelan et al. [16] Canada	Content analysis	YouTube videos	None raised
Lee et al. [17] Korea	Retrospective content analysis and descriptive statistical analysis	HPV website Q & A posts	None raised

### Soundness of research design

The aim or purpose of each study was clearly stated. In three studies, the rationale for undertaking the study was explicitly described as filling a gap in knowledge, however, only two of the six publications provided a robust literature review. All six studies provided details about sampling, data sources, and data collection and analysis procedures that were appropriate to the research questions. Two provided more evidence than the others of how they built in rigor (e.g., inter-coder reliability testing, triangulation of data sources, representative sampling) to ensure the trustworthiness and credibility of their findings. We identified some concerns about selection bias and the representativeness of samples given that blogs and YouTube are, by their search term-based nature, more likely to attract viewers and posters with a vested interest in learning about or expressing particular views and opinions about any given topic. This is significant especially if researchers make a claim of applicability beyond the specific virtual community to the wider public. Such was the case when Keelan and colleagues set out to “map the public debate” over implementation of HPV vaccination by collecting data on a single day from 303 Myspace blogs [15].

Discussions of ontology (the nature of reality) and epistemology (the nature of knowledge) and how these influenced the methodological framework were not taken up by the authors of four of the six studies. This absence, it might be argued, is consistent with a quantitative research paradigm [20] and therefore not considered important (or even relevant) to report in these papers.^d^ The Battles article [14] engaged in a critically reflexive discussion of the methodological issues of conducting internet-based research with youth. The findings reported by Briones and colleagues [19] were framed using the health belief model. None of the other five studies framed findings using a theoretical model.

Some researchers identified practical problems working in a virtual environment and recommended ways that future researchers might ensure the integrity of data collection. For example, Briones and colleagues [19] discovered that if selected videos were not downloaded in their entirety on the designated day of collection, they might be unavailable at a later date for review and analysis.

Overall, the designs of the six selected studies were sound. Most study designs may have been strengthened with greater transparency about building in rigor, explicit caveats about the generalizability of findings beyond the virtual community, and more attention to ethical considerations (to be discussed in greater detail later).

### Social media as a data collection source

This metamethod of selected HPV vaccine studies indicates that researchers have differing conceptualizations of social media. Briones and colleagues [19] were the only researchers who used the term *social media* to name the virtual space used to explore online coverage of HPV vaccines. Although the other selected articles did not use this term in describing their own studies, Briones et al. used this term when citing three of these selected studies. They referred to the study by Keelan and colleagues [15] as research that used “the online social media sphere” to explore public discourse about HPV vaccination [19]. When describing the studies conducted by Ache and Wallace [18] and Keelan et al. [15] they referred to YouTube as “another social media channel” [19]. How researchers define (or fail to define) this emerging research space and the virtual communities that use them may provide insight into how researchers used these platforms to collect data.

In four studies, data collection was uni-directional (meaning that researchers mined user-posted data) while authors simultaneously pointed out the multi-dialogical character of social media. Ache and Wallace referred to YouTube as an example of the increasingly popular “participatory Internet sites” [18]. Briones et al. also noted that YouTube’s “free service, easy accessibility, and opportunities for sharing” have extended its original purpose from a venue for sharing consumer-generated content to an “entertainment destination” [19]. Similarly, Keelan et al. note the expressed concerns of health professionals about the use of internet sites such as YouTube to share information about the risks and benefits of immunization [16]. They described this form of technology as a way to “broadcast alternative viewpoints and influence public debates […] and disseminate medical information unimpeded by the expert medical community” [16]. Although in each case researchers recognized that internet sites were a way to engage in dialogue with the public, this belief did not translate into a dialogical engagement with virtual users in the research process.

Lee and colleagues referred to the website they created as a HPV “public service” portal for users seeking medical and health-related information of a confidential nature [17]. The focus of the study was on quantifying the types of questions posted and the demographics of the users rather than on understanding context or engaging in meaningful dialogue between the physician-researchers and website users.

Battles [14] conducted the only study in this sample that engaged in a two-way dialogue with users over time about HPV vaccination. Described as an exploratory virtual ethnography, the researcher performed a content analysis of discussion threads posted by female participants on an internet message board in response to the question, “Are you getting the HPV vaccine?” She referred to internet-research as a “relatively new and growing field” and online communities as “rich sources of qualitative data” [14]. This explicit focus on qualitative data (and the implicit attention to subjectivity) may account for the interactive approach to research.

### Ethical issues

Only one of the six articles (Battles [14]) explicitly discussed the ethical considerations involved in conducting research on a social networking site. The researcher sought approval from her institutional research ethics board and obtained consent from the participant members of the virtual community. Battles flagged a series of ethical concerns about using social media as a data source for health research (e.g., privacy and self-disclosure; confidentiality; informed consent; what constitutes a community and who needs to give consent; trustworthiness of data; dissemination of data; use of data in knowledge translation) [14]. The researcher provided detailed information about how to navigate ethical issues and highlighted the problems of using social media as a data source, especially where youth may be involved.

Lee and colleagues [17] created a question and answer interface allowing users to post private or public questions. They explained their decision to develop and use their website as a data source for exploring public knowledge about HPV transmission and infections as well as attitudes toward HPV vaccination by saying that a “well-developed information technology and internet infrastructure” would enable them to engage in “public conversation about sexual behavior [that] is traditionally considered taboo” in Korea, a “Confucianism-based culture” [17]. The Q & A webpage encouraged users to post public rather than private questions as a way to “encourage information sharing by visitors” [17]. With this juxtaposition of notions of private and public, it is unclear how Lee and colleagues construed the community of users or the social boundaries in this space. There was no indication that consent was requested nor given or that users were aware that posted questions and demographic information were being mined for research purposes by the website physician-researchers.

Of the remaining four studies [15, 16, 18, 19], there was no mention that the researchers sought ethics approval from an institutional research ethics board. There was no evidence that the researchers sought the permission of the creators of Myspace or YouTube or the web service provider to use their sites for research purposes.

## Discussion

Three main findings emerged from this metamethod: (a) missed opportunities for taking full and positive advantage of the social in social media; (b) differing assumptions about the social boundaries of public and private communication that occurs in virtual communities; and (c) the ethical dilemmas that emerge from these assumptions.

### Missed opportunities

Some health researchers appear to be missing opportunities to capitalize on the social in social media. Social networking sites are, by design, a way to engage, collaborate, and interact with others in two-way or multi-dialogical conversations. Myspace originating in 2003, Facebook in 2004, YouTube in 2005, and Twitter in 2006 are common forms of social media for creating, sharing, and commenting on information, including health information. All selected studies took advantage of the accessibility of data that social media affords however only two took advantage of the unique opportunity to engage in dialogue with users in an established virtual community. One was Battles’ [14] cyberethnographic study; the other was the study by Lee and colleagues [17]. The latter study might be classified as interactive in that it encouraged users to post questions and receive responses. The authors drew conclusions about what type of information on HPV and HPV vaccines people sought when they consulted an internet site about HPV. However, we cannot know if posted questions represented an unknown piece of information crucial to HPV vaccination decision-making or a simple curiosity. It is unclear whether analyzing questions can adequately reveal a population’s understanding of and beliefs about HPV infections and HPV vaccination. With such specious assumptions, the value of the findings is questionable.

The remaining studies relied on traditional data collection techniques to generate findings. Data were analyzed using positive vs. negative scoring systems (sometimes including an ambivalent or neutral category) to describe HPV awareness and attitudes toward HPV vaccination. Generally the study designs were sound and produced credible findings about attitudes toward HPV vaccination. What was unclear was how valuable this knowledge is to those of us interested in developing public health policies and interventions. While the statistical surveillance used in the majority of these studies offers some sense of users’ opinions, the scoring systems strip data of the context that makes these beliefs and opinions meaningful and fails to probe users’ understanding of HPV vaccines that underpins the posts. Moreover, statistical analysis of such data that intends to measure trends and patterns (in a process similar to conducting a survey) does so without the benefit of randomized sampling and other design elements for ensuring generalizability or reproducibility of findings.^f^

The tone and quality of discourse fluctuates in dynamic ways across various virtual communities. Health researchers must be cautious in making assertions about the wider population based only on a website’s viewers and posters – groups that likely have a vested interest in a given topic and that are sufficiently comfortable with using technology to post their opinions on social networking sites. One cannot assume that data derived from social networking sites are representative of the broader population because of the self-selection bias of users. Thus, the relative value of knowledge generated from such studies seems limited.

We also wonder about the relative value of knowledge produced through uni-directional studies, including some of the selected articles reviewed in this metamethod that were designed to produce objective findings. Can such studies enhance our understanding of users’ subjective opinions, values and choices about an important health concern? Even the relatively well-executed statistical analyses of uni-directionally mined data – as was the case in the majority of the selected studies – cannot render more visible the complexities of HPV awareness or HPV vaccination decision-making. There was no explicit question posed to elicit a response as happens with a survey where parameters and variables are defined. This makes it unclear what precise stimulus participants were responding to when they posted a “thumbs up/thumbs down” or “like/unlike” to videos or blogs. A positive response could just as easily reflect appreciation of visual representations as it might a positive reaction to the content. Therefore, research that investigates public awareness of HPV and HPV vaccination (and other health issues) must reflect this complexity in ways that go beyond a tabulation of binary responses.

This metamethod suggests that traditional data collection techniques may require modification for use in a virtual environment. Moreover, traditional data mining fails to take full advantage of the dialogical nature of social media. Researchers who make full use of this feature might generate richer understandings of the complexities of users’ knowledge of HPV infections and HPV vaccination. Collaborative engagement with users, as in the Battles cyberethnography, acknowledges the shared authority and expertise of the researcher and the researched. At the risk of re-entrenching the quantitative-qualitative debate in health research [20, 21], we are left to wonder if it might be more valuable to embrace and explore the subjectivity of communication in online communities rather than hold to a research paradigm that seeks an objective truth [22].

### Conceptualization of virtual communities: the public versus private binary

According to the Association for Internet Research, the internet is “a social phenomenon, a tool, and also a (field) site for research” [23]. The same might be said of social media. The rapid growth of social media over the past decade has been nothing short of phenomenal. Users post commentary and share information about a range of topics. Organizations are increasingly using Twitter, Facebook, and other social networking sites to perform health research [24]. What is less clear is how researchers conceive of this field site and if these conceptualizations are consistent with those held by those who operate in these spaces.

“Networked publics,” a term coined by boyd and Marwick, refers to “(1) the space constructed through networked technologies and (2) the imagined community that emerges as a result of the intersection of people, technology, and practice” [25]. When considering the specific case of HPV awareness and HPV vaccination, social networking sites attract youth and those who make decisions for youth. Medical experts and others with varying levels of knowledge may also participate in these sites with a goal of shaping public opinion, effecting social change, or promoting a particular public health intervention. If the research goal is to map public awareness and attitudes about HPV infections and HPV vaccination by collecting data from networked publics, the methodological approach must reflect the complexity of knowledge consumption and decision-making. These complex processes require fact-gathering, critical evaluation of evidence, and thoughtful weighing of pros and cons in the context of one’s own life. This means taking advantage of social media as more than a tool for mining data while still utilizing it as a field site for research.

As with accessing any research field site, attention must be paid to understanding the social boundaries operating in that physical or virtual space. To quote boyd, “[i]t’s about a collective understanding of a social situation’s boundaries and knowing how to operate within them” [26]. Community members may post information or commentary in a public virtual space while maintaining divergent expectations for how those postings are used by others. The Association of Internet Researchers Ethics Working Committee writes:

Individual and cultural definitions and expectations of privacy are ambiguous, contested, and changing. People may operate in public spaces but maintain strong perceptions or expectations of privacy. Or, they may acknowledge that the substance of their communication is public, but that the specific context in which it appears implies restrictions on how that information is – or ought to be – used by other parties [23].

The Committee concludes that the “[s]ocial, academic, or regulatory delineations of public and private as a clearly recognizable binary no longer holds in everyday practice” [23].

### Ethical issues

How do health researchers engage in ethical research in virtual communities? This important question has been taken up by individual scholars [22, 24–26] and by the Association for Internet Researchers [23]. The answer seems to pivot on the ambiguity about social boundaries and the application of the public/private binary in networked publics. This metamethod suggests that some health researchers are not addressing these concerns. Five of the six studies were based on the assumption that social media was an accessible source of unfiltered information about public attitudes toward HPV vaccination as these studies aimed to characterize public attitudes about HPV and HPV vaccinations. The public accessibility of data seemed to justify its use even if no consent was obtained from users or service providers that data were being mined for research purposes. However, boyd and Crawford assert that “[j]ust because it [data] is accessible does not make it [using the data] ethical” [22].

Traditionally, if research involved human subjects, it had to undergo ethics approval. Since the adoption of the Nuremberg Code in 1947, research ethics guidelines have required that human subjects must be protected in health research. The Code was drafted in reaction to the ethical implications of the Nazi medical experiments and emphasized the importance of informed consent [27]. However, the Association of Internet Researchers contends that the concept of human subjects is not well suited to research that does not involve biomedical interventions or research that interacts indirectly with people through archival materials or other published texts [23]. Instead, the Association recommends focusing on concepts of harm, vulnerability, personally identifiable information, and other such concepts [23]. This recommendation is consistent with the Tri-Council Policy Statement 2 (TCPS2),^g^ which sets out three principles for ethical decision-making: concern for welfare, respect for person, and justice [28].

The potential for harm is covered under the principle *concern for welfare*, or “the quality of that person’s experience of life in all its aspects” [28]. Under this principle, researchers have an obligation to both assess and minimize risk while balancing risks and potential benefits. Accordingly, those individuals whose data are included in a study have a right to make the final judgment about the acceptability of this balance. It cannot be assumed that using social media means that individuals have forfeited their right to make this judgment. In five of the six studies, individuals were not informed and did not consent to having their data collected for research purposes. Nor was there evidence that the researchers considered the potential harm to individuals whose data were used in a way they might not have intended or imagined. In the sixth study, Battles [14] obtained informed consent acknowledging that attempting research in a virtual community is difficult because participants may wish to remain anonymous to the researcher and because the transient nature of social media participation may make it impossible to track users. This prompts the question: does mining data without informed consent constitute a risk of harm? This question seems especially salient given the sensitive (even taboo) nature of cultural attitudes toward HPV infections and vaccinations in some networked publics where data were mined.

The second principle identified in the TCPS2, *respect for person*, involves the moral obligation to respect and protect the autonomy of research participants as well as those individuals whose data are used for research purposes [28]. Obtaining free, informed, and ongoing consent is an accepted mechanism for doing this. The TCPS2 devotes minimal attention to research in virtual settings, equating these spaces with observational studies conducted in natural settings “where people have a reasonable or limited expectation of privacy” [28]. In these situations the researcher must justify the exception to the requirement to obtain consent. Our metamethod indicates that ethics approval was only sought for the cyberethnography, leading us to think that researchers involved in the other five studies assumed that virtual communities were public spaces where users had no little or no expectation of privacy. There was an absence of discussion about the potential privacy incursion even when users’ comments were cited. Failing to engage users about the subsequent use or interpretation of data they posted may have unintended negative implications. For instance, Ache and Wallace directly quote negative and positive viewer-posted comments about HPV videos appearing on YouTube [18], making it possible to identify the accounts of those individuals whose comments were cited.

When researchers publish users’ verbatim comments in scholarly articles, it is possible for readers to reproduce the quotations in a search engine that can link them to an individual’s account on the sampled social media website. Using this search method for an article that quotes YouTube comments, article readers can glean information about a willing or unwilling study participant by reviewing the “about” information in his or her public profile or by analyzing the videos followed by the individual. The potential exists for article readers to further identify such individuals by using the social media website as a platform to contact the person through messaging or review his or her posted content, which may include personal videos or photos.

On the other hand, researchers’ efforts to protect online study participants by not presenting direct quotations as part of the data set can negatively affect the trustworthiness and credibility of results. The study that paid most attention to the ethical protection of users (Battles [14]) provided little evidence on which we could evaluate the quality of design or assess the rationale behind interpretations and conclusions about users’ understanding of HPV vaccination. To better maintain the principle of respect for person while presenting verifiable data, we recommend that researchers paraphrase users’ comments. If studies presented a topical but fictive sample quotation alongside the resultant paraphrase, then readers could more easily evaluate the credibility of the results by assessing how meaningfully authors can capture opinions in paraphrase.

*Justice* is the third principle listed in the TCPS2 and refers to fair and equitable treatment [28]. Power, especially the power differential between the researcher and the researched, must be considered. Where the power imbalance is abused, there is a significant threat to justice and the potential for harm [28]. In many jurisdictions, youth are considered vulnerable and deserving of special consideration because of their limited access to rights, opportunities and power [28]. Youth are both eligible candidates for HPV immunization and a population that is technologically capable of using social networking sites to seek and share health information [7]. Therefore it is reasonable to assume that youth may make up a portion of the users drawn to HPV information content. Youth and other vulnerable populations might not understand research in the same way and to the same degree as a researcher, particularly when privacy and self-disclosure may have implications beyond the immediate moment. Some cultural, social and religious groups should also be afforded special attention to ensure they are treated justly in research. This might be said of Korean users who visited the Q & A website that was established precisely because conversations about sexual behavior were considered taboo in a “Confucianism-based culture” [17]. With the exception of the Battles study [14], there is no evidence that researchers considered the expressed needs and concerns of the participants whose data they collected or whether mining data might harm potentially vulnerable users. Researchers are also at risk of misinterpreting or misrepresenting users’ comments. In the absence of dialogue it is difficult to assess if the participants would evaluate the researcher findings as accurate interpretations of their experience.

Given the relative newness of virtual communities, researchers and institutional ethics review boards must work together to develop expertise in evaluating the design of studies undertaken with virtual communities. Currently, ethics board review and approval is required only for research with “live” human subjects and is not required for research in traditional public spaces. As Zimmer points out, institutional ethics review boards lack the expertise to consider the potential privacy incursions or risk of harm conducted on social networking sites [24]. The Association for Internet Research developed some guidelines in 2002 with a more recent revision in 2012 [23]. However, if this metamethod is any indication, these ethical dilemmas and the guidelines for conducting ethical research in social networking sites are not widely known or implemented. Indeed, when we presented our initial findings at an academic conference in 2012, audience members dismissed the ethical concerns we identified calling up the public square argument. The public square argument used by research ethics boards assumes that ethics approval is not required when research involves the observation of people in traditional public spaces. When this guideline is extended to online spaces, it is used to assert that that individuals posting data on a social networking site are inviting public engagement and thus cannot reasonably hold an expectation of privacy. The users’ intended use of the public space (i.e., to share information and engage in public discussion) does not reasonably extend to consent for another purpose such as research. The public square argument does not address the concern that data are being used for a purpose other than what the users may have intended with an attendant risk of harm.

The application of the core principles of concern for welfare, respect for person, and justice must extend to networked publics (as both virtual spaces and the users who occupy them) with explicit justification provided for not seeking informed and ongoing consent if social boundaries are unclear. This ethical approach to engaged and collaborative research will help to build and maintain the trust of participants and the public in the research process and ensure that the benefits of knowledge production are shared [28]. These findings seem especially relevant considering the public backlash against Facebook following their mood manipulation study [29].

### Limitations

The findings should be interpreted with caution considering the small sample size. The synthesis of the findings based on six studies is descriptive and may not necessarily reflect all methodological or ethical considerations. Because our inclusion criteria were limited to HPV vaccination studies, findings may differ in other health studies using social media.

## Conclusions

Our goal was to explore the soundness and adequacy of research methods used in virtual communities with a view to identifying problems that might be avoided in designing a project about HPV infections and HPV vaccination. This metamethod revealed a disconnect between the dramatic growth in the number and variety of social networking sites, the lack of clarity about social boundaries of private and public in virtual communities, and how health researchers’ practices are changing (or not) to reflect the unique characteristics of this new environment.

Social media can be a go-to place for tuning into public discussion about health issues [4], and sharing and responding to information on YouTube, blogs and message boards [3]. Although users of social media generally perceive it as an interactive platform for engaging with others [7], we found only modest evidence that health researchers are taking advantage of the richness of social media as a data source for studying public understandings about HPV and HPV vaccination. As such, the opportunities for using social media for health research do not appear to be fully realized [22]. Moreover, the accessibility of data on social networking sites is attractive to health researchers who are able to mine data in ways that may be considered by some to be unethical and exploitative.

Two concluding thoughts will guide our next steps, and might serve as actionable messages for others intending to design a study with networked publics. First: be clear about the social boundaries of the intended virtual community and how these may influence users’ perceptions of public and private. Where boundaries are ambiguous, we intend to err on the side of concern for welfare, respect for person, and justice in conducting health research, especially where there is a potential for engagement with youth or vulnerable populations. Second: we intend to take advantage of the dialogical character of social media by collaborating with networked publics to generate health knowledge that reflects both the expertise of researchers as well as the expertise and embodied experiences of the public to whom we are accountable.

## Endnotes

^a^We name these social media platforms to make our meaning clear for the 2014 reader knowing that the continuum of interactivity and content in different social media types will render these examples less relevant or meaningful over time.

^b^As Bondas and Hall point out, one of the limitations of the metamethod (in general, and with this study specifically) is that data collection and analysis of the research design is confined to what appears in the primary journal article and not necessarily what went on behind the article [9]. Researchers may focus greater attention to presenting findings rather than describing in detail the methodological assumptions and methods. This may be due, in part, to journal-imposed restrictions on the length of a manuscript or the emphasis on outcomes-oriented research or producing evidence to guide health policy, programs or practice.

^c^Reasons for excluding studies included: not primary research reports, the study mentioned HPV but focused on other vaccination programs (e.g., H1N1, influenza, measles); social media referred to all types of news coverage and communication.

^d^Quantitative research grounded in a positivist paradigm is less likely to be reflexive about and explicitly document epistemological stance than qualitative research where revealing these deep structures is considered central to establishing trustworthiness of findings.

^e^For more detailed information on our findings, contact the corresponding author.

^f^Problems with reproducibility may be heightened in social media research because of the fluid character of platforms themselves. Researchers’ (and users’) conceptions of what constitutes social media will shift with the ever-expanding technologies and practices.

^g^TCPS2 is a Canadian policy that governs research conducted with human participants. Although institutional review boards in the United States and elsewhere may have different guidelines and practices, the history leading up to the development of the guidelines is shared [30].
